# Peer Inclusion in Interventions for Children with ADHD: A Systematic Review and Meta-Analysis

**DOI:** 10.1155/2018/7693479

**Published:** 2018-03-18

**Authors:** Reinie Cordier, Brandon Vilaysack, Kenji Doma, Sarah Wilkes-Gillan, Renée Speyer

**Affiliations:** ^1^School of Occupational Therapy and Social Work, Curtin University, GPO Box U1987, Perth, WA 6845, Australia; ^2^College of Healthcare Sciences, James Cook University, 1 James Cook Drive, Townsville, QLD 4811, Australia; ^3^School of Allied Health, Australian Catholic University, P.O. Box 968, North Sydney, NSW 2059, Australia; ^4^Department of Special Needs Education, University of Oslo, Postboks 1140 Blindern, Olso 0318, Norway; ^5^Department of Otorhinolaryngology and Head and Neck Surgery, Leiden University Medical Centre, P.O. Box 9600, 2300 RC Leiden, Netherlands

## Abstract

**Objective:**

To assess the effectiveness of peer inclusion in interventions to improve the social functioning of children with ADHD.

**Methods:**

We searched four electronic databases for randomized controlled trials and controlled quasi-experimental studies that investigated peer inclusion interventions alone or combined with pharmacological treatment. Data were collected from the included studies and methodologically assessed. Meta-analyses were conducted using a random-effects model.

**Results:**

Seventeen studies met eligibility criteria. Studies investigated interventions consisting of peer involvement and peer proximity; no study included peer mediation. Most included studies had an unclear or high risk of bias regarding inadequate reporting of randomization, blinding, and control for confounders. Meta-analyses indicated improvements in pre-post measures of social functioning for participants in peer-inclusive treatment groups. Peer inclusion was advantageous compared to treatment as usual. The benefits of peer inclusion over other therapies or medication only could not be determined. Using parents as raters for outcome measurement significantly mediated the intervention effect.

**Conclusions:**

The evidence to support or contest the efficacy of peer inclusion interventions for children with ADHD is lacking. Future studies need to reduce risks of bias, use appropriate sample sizes, and provide detailed results to investigate the efficacy of peer inclusion interventions for children with ADHD.

## 1. Introduction

Attention-Deficit Hyperactivity Disorder (ADHD) is the most prevalent neurobehavioural disorder affecting school-aged children [1]. Impaired social functioning is regarded as one of the core deficits for children with ADHD [2, 3]. Individuals with ADHD frequently present with deficits in the following executive function domains: problem solving, planning, flexibility, orienting, response inhibition, sustained attention, and working memory [4]. They also experience affective difficulties, such as motivation delay and mood dysregulation [4]. These difficulties appear to form the basis of the social skills problems in children with ADHD [5, 6].

Quality friendships are important for children's development and serve as a protective factor for those at risk for current and future difficulties [7]. While having friends has been found to be developmentally advantageous throughout the lifespan [8], more than 50% of children with ADHD experience peer rejection from their classmates [3, 9]. Typically developing peers often describe children with ADHD as being annoying, boisterous, irritating, and intrusive [6]. Furthermore, the interpersonal relationships of children with ADHD are frequently characterised as being negative and conflicting [3, 10]. Children with ADHD are likely to have difficulties in establishing and maintaining satisfying interpersonal relationships as a result of difficulty with cooperative play with peers, perspective taking, responding to social cues, and self-regulation, placing them at higher risk of social isolation [11].

There is a large body of empirical research that demonstrates that children with ADHD experience pervasive social difficulties that can cause social maladjustment in adolescence and adulthood [3, 7, 12]. Impairments in social functioning can lead to school dropout, academic underachievement, low self-esteem, and troublesome interpersonal relationships with family members and peers [13]. As a result, children with ADHD are at greater risk of developing adverse problems in adolescence and adulthood, including anxiety, depression, aggression, and early substance abuse [3].

There is much debate surrounding the causes of social skills deficits in children with ADHD. Some researchers theorise that the social difficulties of children with ADHD are a result of having limited knowledge of age-appropriate social skills, proposing that the social skill deficits are caused by* deficits in skill acquisition *[3]. Other researchers have drawn from the well documented cognitive model of ADHD to explain the mechanisms underlying social skill deficits in children with ADHD [52]. In this conceptual model, Barkley [52] concluded that children with ADHD possess adequate social skills but fail to apply them in specific social situations; thus their social skills deficit is a result of a* performance deficit*.

Recent reviews conclude that* performance deficits *are the likely cause of social problems in children with ADHD [99, 100]. Children with ADHD appear to possess age-appropriate social skills; however they fail to* apply* this knowledge to functionally interact with others [101]. This lack of application of knowledge is likely due to a range of cognitive and affective difficulties, where children with ADHD may demonstrate disproportionate emotional reactions and decreased perspective taking and forethought, impacting their ability to apply the necessary skills during spontaneous social interactions with peers [52].

Several clinical practice guidelines, including those of the National Institute for Health and Clinical Excellence (NICE) in the United Kingdom, have concluded that nonpharmacological interventions are a necessary component when treating children with ADHD [102]. The effectiveness of using nonpharmacological interventions, such as parent training (PT), cognitive-behavioural therapy (CBT), social skills training (SST), school-based interventions, academic interventions, and multimodal treatment, has been reviewed for children and adolescents with ADHD [102–104]. Although SST has been reviewed extensively, the core components of psychosocial treatment, such as the use of peers in the interventions aimed at improving social skills, have not been systematically investigated for children with ADHD.

Peers are commonly included in psychosocial interventions for children. Peer inclusion interventions are often coupled with psychoeducational interventions such as parent training and/or school-based interventions where teachers implement daily report cards and behaviour response-token strategies [104]. Peer inclusion interventions can also be implemented within the context of a summer treatment program where a range of different psychosocial interventions are conducted to improve ADHD symptoms, social functioning, and overall impairment [105]. Peer inclusion in interventions is postulated to have multiple benefits. Including peers in interventions may motivate children to participate and allow the intervention to be conducted in group settings, enhancing the feasibility of the approach [46, 55]. Moreover, including peers in interventions has the possibility of improving intervention outcomes [106]. From a social learning theory perspective, children are presented with frequent opportunities where social skills, behaviours, and consequences are modelled during group interactions [107]. Across the literature on psychosocial interventions for children with developmental disorders, the types of peer inclusion have been broadly described and categorised as follows: (a) peer involvement, (b) peer mediation, and (c) peer proximity.

Peer involvement has been most commonly used in SST and summer treatment programs (STP) interventions for children with ADHD. Peer involvement is most commonly characterised by interventions where participants facilitate each other's learning. Therefore, the number of opportunities to reinforce and practice target skills is increased, enhancing the success of treatment outcomes. The children are taught social interaction strategies such as sharing, helping, prompting, instructing, or praising [108]. However, peers included in these interventions often include children with similar diagnoses and skill difficulty in a group therapy context. Thus, intervention may incorporate facilitator-led role-plays and interactions, where the focus is on increasing social skills through instructions during peer-to-peer interactions [53, 61, 66, 69].

Peer-mediated intervention involves an extension of peer involvement as the peer is a key component and an active agent of change for the intervention. In peer-mediated interventions, peers are trained to provide instruction and facilitate social interactions with the target child/client [109]. Peer-mediated intervention involves a combination of peer initiation, modelling, prompting, and reinforcing of the desired behaviour [106]. Peer-mediated interventions can be readily incorporated into a child's environment, particularly in inclusive settings, and can support the generalisation of skills across different environments [106]. Peer-mediated interventions are based on the notion that individuals' behaviour is influenced by their peers, an influence that can be both overt and powerful [110]. For these reasons, typically developing peers have been most commonly incorporated into peer-mediated intervention with stringent criteria regarding peer selection [106, 111].

Peer proximity involves carefully selected peers of increased skill, likely without a diagnosis, who are placed in close proximity to the child, such as sitting at the same table in a classroom [112]. Central to both peer-mediated and peer-proximity approaches is the careful and purposeful selection of peers. Commonly used inclusion criteria for peers in both peer-mediated and peer-proximity interventions were as follows: typical social and language development, absence of behaviour difficulties, an interest in interacting with the target child, and regular availability [46, 106, 108, 111]. The direct interaction between the client and their peers, which is the central characteristic of peer-mediated and peer-proximity interventions, has many practical advantages and benefits including fostering inclusion in school settings [111]. An example of such an advantage is the abundance of typically developing peers in schools and the use of a practical approach to provide services to children with additional needs that could lower cost and alleviate pressures on teachers, health professionals, and parents [113].

A peer-mediated approach is the most empirically supported model of social skills interventions for children with Autism Spectrum Disorders (ASD) [108]. However, further research is required to strengthen the evidence base of the use of peers in social interventions for children with ADHD. Similar to children with ADHD, children with ASD experience significant social skills impairments. Training peers to support social skills development in target populations is regarded as ecologically valid for children and has the potential to address the problem of limited generalisability of treatment effects in adult mediated interventions [114]. As such there is a need to conduct a systematic review to examine the effectiveness of peer inclusion in interventions aimed at improving the social functioning for children with ADHD.

This systematic review aimed to examine the efficacy of peer inclusion in interventions targeting the social functioning of children with ADHD. To capture the use of peers in interventions in the existing literature and for the purpose of this systematic review, peer inclusion interventions were defined as interventions that reported peer involvement, peer mediation, or peer proximity. We also aimed to identify and summarise the key characteristics of a range of peer inclusion interventions, which will be used to analyse the feasibility of using peers in treatment interventions for ADHD. Furthermore, we conducted a meta-analysis to examine the significance of improvements and effect sizes of peer inclusion interventions designed to improve the social functioning of children with ADHD. The manner in which improvements and effect sizes varied between specific treatment approaches was also examined.

## 2. Method

The methodology and reporting of this systematic review were based on the PRISMA statement (see Supplementary Table  1). The PRISMA statement checklist covers areas considered necessary for the transparent reporting of systematic reviews in areas of health care [115].

### 2.1. Information Sources

To locate eligible studies, the fifth author conducted literature searches across four electronic databases between November 4 and 7, 2016. The searched databases included the following: CINAHL, PsycINFO, Embase, and Medline with the following dates of coverage 1937–2016, 1887–2016, 1902–2016, and 1946–2016, respectively. Supplementary search approaches such as checking reference lists were also used to identify studies.

### 2.2. Search Strategy

Studies were identified through the following procedure during the initial and updated searches. First, an electronic database search was conducted using CINAHL, PsycINFO, Embase, and Medline. Two categories of search terms (e.g., Mesh and Thesaurus terms) were used in combination: (1) disorder (Attention-Deficit Hyperactivity Disorder (ADHD), Attention-Deficit Disorder (ADD), and Attention-Deficit Disorder with hyperactivity) and (2) psychosocial interventions (peer, friend, friendship, buddy, playmate, group therapy, group intervention, group role-play, play group, play therapy, play treatment, play intervention, camp(s), school-based, play-based intervention, psychosocial, social skills, SST, social groups, social behaviour/behaviour, and group counselling). Limitations applied to the search included subject age (preschool child [2–5 years], child [6–12 years], and adolescent [13–18 years]), English language, and humans. The full electronic search strategy used for one of the major databases (Embase) is reported in Table 1. Using subheadings, free text searches were also conducted for all four databases for studies published within the year prior to the search. The search terms and limitations for the free text searches are also described in Table 1.

### 2.3. Inclusion/Exclusion Criteria

The following criteria for inclusion were applied: (1) children and/or adolescents had to have a primary diagnosis of ADHD according to the* Diagnostic and Statistical Manual of Mental Disorders 3rd Edition *(*Revised, *DSM-III-R) or* Diagnostic and Statistical Manual of Mental Disorders 4th Edition *(DSM-IV) criteria; (2) studies included a control group; (3) the interventions included peers; (4) the treatment content focused on social functioning; and (5) the treatment outcome could be related to the peer inclusion intervention. Multimodal intervention programs in which the peer inclusion intervention was part of a variety of empirically based behavioural components were included if results can be extrapolated to provide insight into the value of including peers as a core variable. These criteria were selected to identify peer inclusion intervention studies that would be classed as either level II or III on the National Health and Medical Research Council (NHMRC) Hierarchy of Evidence [116]. The NHMRC Hierarchy of Evidence was developed by the Australian NHMRC to rank and evaluate the evidence of healthcare interventions [116]. According to the NHMRC Hierarchy of Evidence, level I studies are systematic reviews of randomized controlled trials (RCTs), level II studies are a well-designed RCTs, and level III studies are, for example, quasi-experimental designs without random allocation. Studies with level III evidence were included as it was unlikely that a search limited only to level II studies would identify all required studies to review the literature.

### 2.4. Systematic Review

#### 2.4.1. Methodological Quality

The NHMRC Evidence Hierarchy “levels of evidence” [116] and the Kmet appraisal checklist [117] were used to assess the methodological quality of the included studies. Kmet has a three-point ordinal scoring system (yes = 2, partial = 1, and no = 0) that provides a systematic, reproducible, and quantitative means of simultaneously assessing the quality of research encompassing a broad range of study designs [117]. The total Kmet score can be converted into a percentage score, with a Kmet score of >80% considered strong quality, a score of 60–79% considered good quality, a score of 50–59% considered adequate quality, and a score < 50% considered to have poor methodological quality.

#### 2.4.2. Data Collection Process

A data extraction form was created to extract the data within the included studies. We extracted the data under the following categories: participant diagnosis, control group, age range, mean and standard deviation, inclusion criteria, treatment condition, outcome measures, treatment outcomes, peer/parent/teacher components, skills taught, medication use, method and level of evidence, use of blinding and randomization, and methodological quality (using Kmet).

#### 2.4.3. Data Items, Risk of Bias, and Synthesis of Results

During data collection, data points across all studies were extracted using comprehensive data extraction forms. During this process, risk of bias was assessed at an individual study level during the Kmet rating [117]. Data was then extrapolated and synthesised into a number of categories: participant characteristics, inclusion criteria, treatment conditions and outcomes, components of studies, components of the interventions, and methodological quality. The principal summary measures to assess treatment outcomes were effect sizes and significance of data. We only analysed the effect sizes of the social skills outcomes for the peer inclusion interventions, as the focus of this review was on the use of peers to facilitate social skills development. Interrater reliability for abstract selection and Kmet ratings were established by two independent assessors based on Weighted Kappa calculations. There was no evident bias in scoring study quality and extractor bias of the reviewers conducting this systematic review, as neither reviewer has formal or informal affiliations with any of the authors of the published studies included.

### 2.5. Meta-Analysis

#### 2.5.1. Data Analysis

Data was extracted from the relevant studies in order to compare the effect sizes for the following: (1) pre-post measures of social skills using peer inclusion interventions and (2) mean difference in social skills measures from pre to post between peer inclusion interventions versus comparison controls. Three studies [55–57] were excluded from both analyses as the reported data was not separated from other typically developing peers or other diagnoses. One further study was excluded as true baseline measures could not be provided [63]. To compare effect sizes for both the peer inclusion and comparison group conditions, group means, standard deviations, and sample sizes for pre- and postmeasurements were then entered into Comprehensive Meta-Analysis Version 3.3.070 [118].

Effect sizes were generated in Comprehensive Meta-Analysis using a random-effects model, as it was unlikely that the included studies have the same true effect due to variations in sampling, intervention approaches, outcome measurement, and participant characteristics. Heterogeneity was estimated using the *Q* statistic to determine the spread of effect sizes about the mean and *I*^2^ to estimate the ratio of true variance to total variance. Effect sizes were calculated using the Hedges* g *formula for standardized mean difference (SMD) with a confidence interval of 95% and were interpreted using Cohen's *d* convention as follows: *d* ≤ 0.2 as small; *d* ≥ 0.5 as moderate; and *d* ≤ 0.8 as large [119].

Forest plots of effect sizes for social skill measures' score were generated for the following: (1) pre-post groups for peer inclusion interventions and (2) peer inclusion interventions versus comparison groups. Subgroup analyses were then used to explore the effect sizes as a function of the following: (1) specific type of peer inclusion intervention (peer involvement, peer mediation, or peer proximity) in pre-post group analysis and (2) comparison group type (medication only, treatment as usual, and another therapy) for the peer inclusion intervention versus comparison group analysis.

Publication bias was assessed using Comprehensive Data Analysis software following the Begg and Muzumdar's rank correlation test which reports the rank correlation between the standardized effect size and the variances of these effects [120]. The statistical procedure produces tau which is interpreted as a value of 0 indicating no relationship and deviations away from 0 indicating a relationship, as well as a two tailed *p* value. If asymmetry is caused by publication bias, high standard error would be associated with larger effect sizes. If larger effects are presented by low values, tau would be positive, while if large effects are represented by high values, tau would be negative. Publication bias was also assessed using Duval and Tweedie's trim-and-fill procedure [121]. The procedure investigates the publication bias funnel plot, which is expected to be symmetric. That is, it is expected that studies will be dispersed equally on either side of the overall effect. The trim-and-fill procedure initially trims the asymmetric studies from the right-hand side to locate the unbiased effect and then fills the plot by reinserting the trimmed studies on the right as well as their imputed counterparts to the left of the mean effect size. The program is looking for missing studies based on a fixed-effect model and is looking for missing studies only to the left side of the mean effect.

## 3. Results

### 3.1. Study Selection

A total of 3,395 studies were found across the following databases: CINAHL (280), PsycINFO (1073), Embase (1448), and Medline (594). Only one study was identified through searching of additional sources. The 3,395 studies identified through subject headings and free text searches were screened for duplicate titles and abstracts with 618 duplicates removed. Two researchers reviewed abstracts for inclusion in the review. To ensure rating accuracy, 20 randomly selected abstracts were assessed by both raters to achieve consensus before rating the remaining abstracts. A third researcher (second author) was consulted if agreement could not be reached between the first two researchers to achieve 100% consensus. The agreement (Weighted Kappa) between raters for all abstracts was 0.832 (95% CI 0.5648–1.000). A five-point ordinal scale was constructed to rate abstract eligibility using the five inclusion criteria (described earlier), and abstracts with a score of 4 or 5 were selected for full-text review.

**Figure 1:**
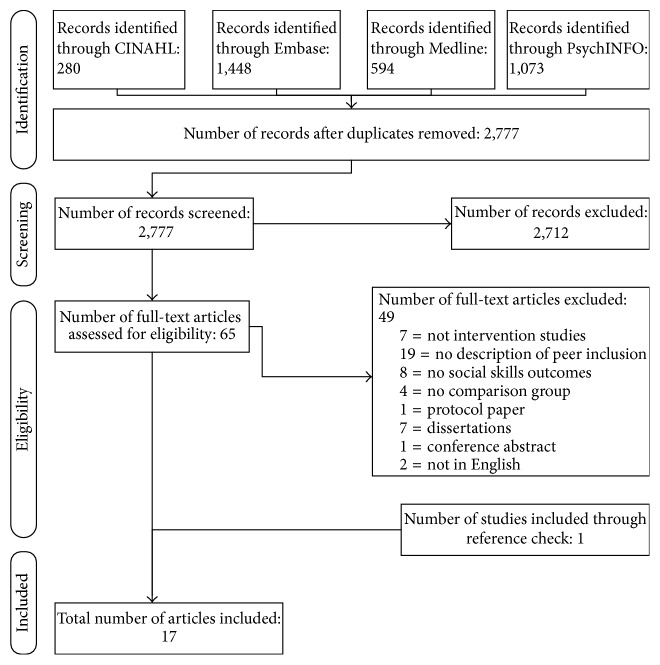
Flow diagram of the reviewing process according to PRISMA [115].

After assessing the abstracts based on criteria created by the research team, a total of 65 studies were identified. Full-text records were accessed to determine if the studies met inclusion criteria. Of these 65 studies, 7 were not intervention studies, 19 did not provide a description of peer inclusion in the interventions with 8 of those studies assessing ADHD symptoms and not social skills outcomes, 8 were peer inclusion studies but did not report social skills outcomes, 4 were peer inclusion studies but did not include a comparison group, 1 was a protocol paper describing an included interventions, and 2 studies were not in English (Figure 1). A list of the studies published in peer reviewed journals that were excluded and reasons for their exclusion are provided in Table 2. Based on the inclusion criteria, 17 intervention studies were selected (see Table 3). All included studies used a controlled design, provided a detailed description of the population, and included the use of peers to facilitate treatment outcomes. The design and rationale of one of the studies [53] were reported in another publication [122]. Therefore both articles were assessed together to maximise data collection.

### 3.2. Description of Studies

The included studies are described in detail in Tables 3–5. The information was grouped and synthesised as follows: peer inclusion intervention studies for children with ADHD (Table 3); intervention components of included studies (Table 4); and methodological quality of included studies (Table 5).

### 3.3. Participants

The 17 studies included a total of 2,567 participants aged between 6 and 16 years with 74% of participants being male. A total of 2,284 participants received a diagnosis of ADHD. Diagnosis was confirmed with various tools based on the international DSM-III-R or DSM-IV with parent and teacher interviews or reports on symptomology. The children with ADHD had the following comorbidities in the included studies: anxiety disorder, affective disorder, tic disorder, depressive disorder, learning disorder, conduct disorder (CD), developmental disorder, and oppositional defiant disorder (ODD), with the exception of Hantson, Wang [59], and Hannesdottir Hannesdottir, Ingvarsdottir [58] that did not report on comorbidities. There was a large variation of sample sizes between the included studies, ranging from 24 to 579 participants (Table 3). Of the included studies, only two trials reported a power analysis to determine a sample size calculation before the start of the trial [64, 68].

### 3.4. Interventions

The 17 studies comprised multiple interventions, including Social Skills Training (SST) [54–56, 59, 60, 67, 68], behavioural treatment [57, 66, 69], behavioural and SST [62, 63], and multimodal behavioural/psychosocial treatment [53, 58, 61, 64, 65]. The interventions involved various components of peer inclusion elements and parents and/or teacher involvement (see Table 4).

### 3.5. Experimental Groups

Ten studies involved child focused SST [54–56, 58–60, 62, 63, 67, 68], with four of these studies incorporating additional parent training [55, 59, 60, 68]. Pfiffner et al. [66] used child focused SST and parent training with the addition of teacher consultation in the experimental group. The MTA trials consisted of child focused SST, parent training, teacher consultation, and classroom behavioural intervention [64, 65]. Haas et al. [57] and Jensen et al. [61] used a behavioural treatment in the context of a summer treatment program; Jensen et al. [61] also included parent training and school-based treatment. Three trials assessed child focused SST and parent training with medical treatment in the experimental group against medical treatment alone [53, 68, 69]. Abikoff et al. [53] also included academic planning skills training and individual psychotherapy. Hannesdottir et al. [58] included additional executive function training via computer-based activities.

### 3.6. Control Groups

Six studies used medications in both experimental and control groups and added one (or more) therapy to medication—thus using “medication only” as the control group [53, 61, 64, 65, 68, 69]. Ten studies utilised either typically developing children or no treatment or assigned participants to waitlist control groups [54–60, 63, 66, 67]. Kolko et al. [62] compared a social-cognitive skills training program against a social activities group where children were merely provided with semistructured opportunities for socialisation rather than a peer-mediated intervention.

### 3.7. Use of Peers

There was great variation between studies as to the degree of detail used in describing and reporting on the characteristics of the included peers. Of the 17 studies, only 3 used non-ADHD diagnosed or typically developing peers to facilitate intervention [55, 57, 63]. Additionally, the involvement of the peers in the intervention varied, with no identified studies reporting detailed involvement of peers to be considered peer-mediated interventions according to our adopted definition. Sixteen studies reported peer involvement [53–62, 64–69] and one study reported a peer-proximity intervention [63].

### 3.8. Risk of Bias in Included Studies

Of the nine RCTs, only two reported generation of random allocation in detail [63, 68]. The other seven RCTs did not report the generation of allocation sequence; therefore the risk of bias was unclear [53, 54, 58, 64, 66, 67, 69]. The MTA trial did report the concealment of allocation, unlike the other trials; thus risk of bias was unclear for those studies. The blinding of participants or clinicians involved in the delivery of interventions is a well-known difficulty [123, 124]. All studies in this review were at risk of bias due to limited blinding of participants. Of the included studies, only two reported blinding for all outcomes [63, 68] and six studies reported blinding for at least one of the outcomes [53, 57, 62, 64, 66, 69]. The studies at high risk of bias due to lack of blinding were as follows: Choi and Lee [54], Frankel et al. [55], Guli et al. [56], Hantson et al. [59], Huang et al. [60], Hannesdottir et al. [58], Jensen et al. [61], MTA Cooperative Group [64], and Shechtman and Katz [67].

Eight studies included data of medicated and nonmedicated children and therefore were at high risk of confounding bias [56–60, 62, 63, 66]. Huang et al. [60] recognised this potential for bias and analysed the impact of drug compliance on results through linear mixed modelling. Waxmonsky et al. [69] conducted a sample size calculation for the primary outcome measure, however not the secondary outcome measures which included the social skills outcome. This may have increased the risk of Type 2 errors as the analysis may not have had the required power to detect trends for all outcome measures. Many of the authors may have had potential invested interest bias, as they have conducted previous research on the topic [53, 55–57, 61, 63–68].

**Figure 2:**
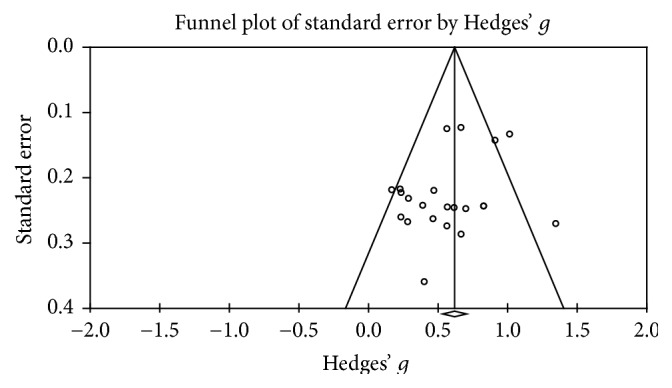
Publication bias funnel plot.

The Begg and Mazumdar rank correlation procedure produced a tau of −0.032 (two-tailed *p* = 0.833), indicating there is no evidence of publication bias. This finding was supported by Duval and Tweedie's trim-and-fill procedure using the fixed-effect model; the point estimate for the combined studies is 0.607 (95% CI: 0.522, 0.692). Using trim and fill these values are unchanged. Under the random-effects model the point estimate for the combined studies is 0.562 (95% CI: 0.431, 0.693). Using trim and fill these values are unchanged. Both of these procedures indicate the absence of publication bias (see Figure 2 for funnel plot).

### 3.9. Methodological Quality

We identified 17 studies published between 1990 and 2016 for children with ADHD. Of these selected studies, nine were randomized controlled trials (RCTs), six were quasi-experimental studies, and two were longitudinal follow-up studies. Of these studies, eleven were classified as level II evidence and six as level III evidence based on the NHMRC Evidence Hierarchy NHMRC [116]. The overall methodological quality of the studies ranged from good to strong with ten studies ranked as good and four as strong according to the Kmet ratings (Table 5). The interrater agreement (Weighted Kappa) for the Kmet ratings was 0.74 (95% CI 0.61–0.86).

### 3.10. Effects of Interventions: Meta-Analysis Results

#### 3.10.1. Effect of Peer Inclusion Interventions on Pre-Post Social Skills Outcomes

**Figure 3:**
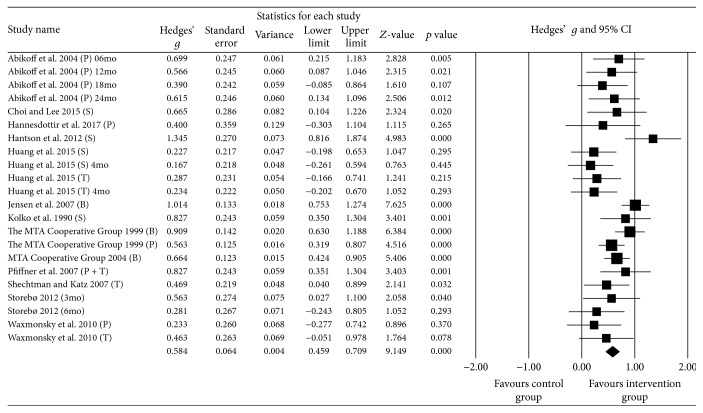
Within intervention group pre-post meta-analysis.* Notes*. Hedges' *g* interpreted as per Cohen's* d* conventions: ≤0.2 = negligible difference, 0.2–0.49 = small, 0.5–0.79 = moderate, and ≥ 0.8 = large.

The pre-post intervention effect sizes for the included studies ranged from 0.167 [60] to 1.345 (large; [59]) (Figure 3). In five of the peer inclusion groups, effect sizes were large, indicating that peer inclusion accounted for a significant proportion of standardized mean difference for these five studies. A significant postintervention between-group effect size total in favour of peer inclusion interventions was found using a random-effects model (*z*(21) = 9.149, *p* < .001, Hedges *g* = 0.584, and 95% CI = 0.459–0.709), indicating moderate improvement in social skills outcomes following peer inclusion interventions. Between-study heterogeneity was significant (*Q*(21) = 40.711, and *p* = .006), with *I*^2^ showing heterogeneity accounted for 48.417% of variation in effect sizes across studies, as opposed to chance.

#### 3.10.2. Effect of Confounds on Pre-Post Social Skills Outcomes

Given the significant heterogeneity, subsequent subgroup analyses were conducted comparing effect sizes between intervention groups to examine variables that could potentially confound social skills outcomes. Comparisons were made based on the following: (a) the presence or absence of parent training and psychoeducation within the interventions; (b) study design (i.e., quasi-experimental design, RCT); (c) methodological quality rating (i.e., good, strong); (d) the presence or absence of blinding for outcome measurement; and (e) the outcome rating respondent (i.e., self-rated, parent rated, teacher rated, and combined parent and teacher rated).

All subgroup comparisons produced a significant result (see Table 6). While analyses based on level of parent involvement, methodological quality rating, and blinding of outcomes measures produced significant results, the differences in effect sizes for subgroups in these comparisons were negligible. Effect sizes for comparisons based on respondent type for outcome measurement ranged from a large positive effect for ratings completed by parents and teachers, to moderate positive effects for ratings completed by teachers or parents or the child with ADHD. Intervention effect favoured RCT studies to a small degree.

#### 3.10.3. Factors Mediating the Intervention Effect

Given the significant results found in all subgroup analyses and similarities in effect sizes for a majority of the comparisons, metaregression was performed to determine if any of the variables contributed as a significant mediator of intervention effect. All variables of the subgroup analysis were entered as covariates in the regression model. Results showed that variable of parents as raters for outcome measurement was the only variable contributing as a significant mediator of intervention effect (*z*(3) = − 2.00; *p* = 0.0457). See Table 7 for full results of the metaregression.

#### 3.10.4. Effect of Peer Inclusion Interventions on Social Skills Compared with Comparison Groups

When comparing peer inclusion interventions and comparison groups, the difference between the pre-post scores for peer inclusion groups and each comparison group type was not significant (*z*(2) = 0.926; *p* = 0.355). Heterogeneity in the included studies was significant (*Q*(21) = 41.032;* p *= 0.006), with *I*^2^ = 48.820% indicating the percentage of variability due to heterogeneity rather than chance. The subgroup analysis indicated when peer inclusion interventions were compared to medication only interventions or treatment as usual, no significant difference was measured (*p* = 0.599 and *p* = 0.644, resp.). A significant but small effect in favour of peer inclusion interventions was measured when compared to other interventions (*z*(6) = 2.440, *p* = 0.015, Hedges *g* = 0.242, 95% CI = 0.048–0.436).

## 4. Discussion

This study aimed to systematically evaluate and analyse the efficacy of peer inclusion interventions in improving social functioning in children diagnosed with ADHD, using systematic review and meta-analysis procedures. The meta-analysis included both RCTs and quasi-experimental studies of peer inclusion interventions, in order to broaden the scope and include all studies which involved peer included elements.

### 4.1. Systematic Review Findings

All but one study by Mikami et al. [63], which employed peer proximity, utilised peer involvement interventions in the form of peer modelling and role-plays. Children were didactically presented with social skills scenarios and were required to teach the other children the correct and incorrect use within a range of contexts. The inclusion of parents and teachers to facilitate generalisability of treatment effects was common for most of the studies included. Eight studies included parent training and psychoeducation of ADHD as an add-on to the peer involvement intervention [53, 55, 59, 60, 64, 66, 68, 69]. Of these eight studies, four also included teacher consultation and daily report cards to increase the behavioural outcomes achieved at school [53, 64, 66, 69].

An important finding is that only 3 of the 17 studies used typically developing peers or peers without a diagnosis for the intervention. This is in stark contrast to findings of a systematic review investigating peer-mediated interventions for children with ASD where 34 of the 42 studies reported using peers with no disability used for the intervention [111]. Moreover, empirical studies have shown the potential negative effect that the involvement of peers with behavioural problems may have on the behaviour of children with ADHD [125–127]. In fact, one study reported that the behaviour of children with the inattentive subtype of ADHD deteriorated following the peer intervention, postulating these children may imitate some of the negative behaviours displayed by other children [126]. The lack of typically developing peers included in the interventions may have reduced potential benefits. There is emerging evidence in literature suggesting that peer inclusion interventions should take the following inclusion criteria for peers into account: typical social and language development, absence of behaviour difficulties, an interest in interacting with the target child, and regular availability [46, 106, 108, 128]. Moreover, it is widely accepted that skill generalisation of social skills is difficult for children with ADHD [129, 130]. As such interventions may have better outcomes when conducted with typically developing peers (including siblings) in the child's natural social environment [113].

Another important finding is that* none* of the studies used peer-mediated interventions, only peer involvement and peer proximity. Given that a peer-*mediated* approach is the most empirically supported model of social skills intervention for children with ASD [108], it is a surprising finding that none of the studies employed peer mediation which, at least in ASD literature, has the best support for improving social functioning. Both children with ASD and ADHD experience significant impairments in social functioning and, given the concomitant presentation of social skills difficulties in these comorbid conditions, a greater overlap in the approach to address the social skills difficulties was expected. The findings of this systematic review point to an urgent need for researchers to give serious consideration to both the inclusion criteria of peers involved in the intervention (i.e., including peers without behavioural problems) and their approach to peer inclusion interventions (i.e., consider incorporating peer-mediated interventions).

A noteworthy limitation of these studies is the paucity of blinding. Without blinding, results may be exposed to a high risk of bias as teachers, parents, and investigators may have vested interests or rate children better due to knowledge of treatment efficacy [124]. Parents are often known as the experts of their children's behaviour; however, they may be inclined to rate their child differently due to a close attachment or a false sense of achievement based on knowledge of treatment. Teachers and investigators may also report incorrect improvements of the treatments if they are aware of diagnosis and/or the treatment itself [123, 124]. The seven randomized controlled trials included in this review are also at high risk of bias due to a paucity of information regarding randomization methods, allocation concealment, and power to detect trends. This is problematic as it limits the ability to blind key stakeholders and determine the necessary number of participants required to detect significance of results [123, 124].

Social skills interventions often have difficulty generalising skills outside of the treatment setting [61, 65]. Peer inclusion interventions aim to address this issue by providing contextual peer relationships to facilitate learning whereby social skills can be further applied to other settings [105]. The included studies did not provide a clear justification as to the efficacy of peer inclusion interventions or the effect of generalisability; however, they do present clear pre-post and follow-up findings of significant improvements in social skills competences and peer interactions through the use of multiple components including peers, parents, and teachers versus waitlist controls or equivalent no treatment controls. Further research should aim to determine the efficacy of treatments where these components are combined and separated to allow for a more clear analysis of the effects of the peer included component of social skills interventions.

### 4.2. Meta-Analysis Findings

#### 4.2.1. Pre-Post Effects and as a Function of Level of Peer Inclusion

We attempted to include as many studies in the meta-analysis as were deemed appropriate, with only four studies being excluded due to inadequate reporting of results or lack of true baseline measurement [55–57, 63]. The meta-analysis revealed a significant improvement in social skills measures and peer relationships for children and adolescents with ADHD when the pre-post scores of participants in the intervention groups were analysed as a whole. However, significant heterogeneity indicated that effect sizes varied across the studies more than would be expected by chance and that these studies cannot be assumed to have been recruited from the same sample. This is unsurprising, given the variation in the treatments including peers and the profile of the participants included in each of the studies.

It is important to note that only peer involvement interventions were included in this meta-analysis. We were unable to make comparisons based on types of peer inclusion, as the single study looking at a peer-proximity intervention did not report true baseline data and hence was not included in the meta-analysis. As such, the literature remains unclear as to the level of peer inclusion required in an intervention in order to maximise the effect of interventions on social skills and peer relationships. Peer proximity should be considered in the future development and evaluation of interventions so that stronger conclusions can be drawn as to the ideal level of peer inclusion for maximising benefits.

The subgroup analysis revealed significant differences between studies based on level of parent involvement, study design, methodological quality, blinding of outcome measures, and the rater completing outcome measurement. However, when effect sizes are compared, negligible differences were identified in three of the comparisons. Interventions involving parents did not differ greatly from interventions without a parent training component, studies with strong methodological quality found similar effects to studies with “good” methodological quality, and blinded outcome measures produced similar results to measures that were not blinded. This lack of conclusive results calls for further research into peer inclusion interventions

The most significant finding of the meta-analysis is the influence that the person chosen to rate outcome measurements can have upon study findings. The person who rated the children's social skills outcomes following peer inclusion interventions showed significance between subgroup differences. Studies that reported on combined parent-teacher ratings showed overall large effect sizes, whereas individual parent, teacher, or child self-ratings demonstrated overall moderate effect sizes. Furthermore, using parents as raters was found to significantly mediate the intervention effect. Careful consideration should be given to measurement selection clinically, and in future studies of peer inclusion, such that observations from a variety of raters are considered and interpreted in the light of these results.

#### 4.2.2. Effects as a Function of Treatment Group Type

Overall, the peer inclusion interventions did not significantly differ from the three comparison group types. The subgroup analysis showed that peer inclusion interventions were more effective in improving social skills and peer relations than comparison interventions for children and adolescents diagnosed with ADHD, but the effect size was small. However, the heterogeneity indicates that participants in these studies cannot be assumed to be drawn from the same sample, suggesting that peer inclusion interventions may not result in better social skills outcomes when compared with other social skill interventions in children and adolescents with ADHD. Further studies are needed to establish the generalisability of the results of this subgroup analysis.

In contrast, the question of whether peer inclusion interventions were more efficacious when compared with other psychosocial and behavioural therapies and pharmacological treatment could not be determined. Results from the meta-analysis revealed that the subgroup overall effect size for medication only as comparison group slightly favoured medication over peer inclusion interventions. Conversely, when looking at the subgroup of peer involvement interventions that were compared to treatment as usual comparison groups results slightly favoured peer inclusion interventions. However, for both subgroup analyses the differences were not significant. It is possible that, as previous research suggests, usual course of medication for children and adolescents peer should be used in combination with psychosocial therapies, such as peer-inclusive treatments in order for clear therapeutic gains to be made.

#### 4.2.3. Other Possible Confounds

The large variation in effect sizes of within-groups pre- to posttest comparisons of peer inclusion interventions could be due to the differing length of treatments in the reviewed studies. Interestingly, in the studies with large effect sizes (e.g., [59, 64]) the treatment programs which included peer involvement interventions were intensive and/or involved long periods of treatment, whereas the study with the smallest effect sizes [69] was designed to be less intensive, replicating an outpatient model in order to curtail the need for extensive involvement of mental health professionals. This indicates that length and intensity of peer involvement intervention could be a confounding factor on posttreatment outcomes. Future research with matched participants receiving varying lengths and intensity of peer involvement interventions are needed to investigate whether length and intensity are significant factors in increasing treatment outcomes. Furthermore, inconsistency in effect sizes may be attributed to a number of confounding variables such as the following: (a) the use of different treatment components; (b) large variation of sample sizes across and within studies; and (c) the influence of medicated and nonmedicated data. Including parents and teachers in the interventions was common amongst the interventions reviewed, with eight studies providing parents with training in addition to peer involvement [53, 55, 59, 60, 64, 66, 68, 69]. Four of those studies also included behavioural therapeutic techniques [53, 64, 66, 69]. As such, the addition of these treatment components parallel with peer involvement makes it difficult to isolate the specific effect of peer inclusion in these interventions. Total sample sizes ranged between 24 and 579 participants with only two studies conducting power analyses to determine their appropriate sample size. Medication was a potential confounder for studies where participants' improvements may have been influenced by the use of medication [57, 59, 62, 63]. Some studies assessed medicated and nonmedicated participants within treatment groups but did not control for their potential confounding influence within their analysis [57, 59, 62, 63]. Therefore the use of this confounding data may have significantly impacted the reliability of results for treatment groups attempting to report on the effectiveness of peer inclusion interventions. Comparing several outcomes across a multitude of different treatments may also cause the comparisons to differ significantly with unclear inferences, as was evident in the Kolko et al. [62] and Mikami et al. [63] studies.

## 5. Limitations

The current study underwent a rigorous review process by searching relevant databases, comprehensively screening abstracts between two independent researchers, and ensuring acceptable interrater reliability agreements for study selection and Kmet methodological quality ratings. Despite the care that was taken to reduce bias, this review is subject to a number of limitations. Every study was at a risk of bias due to inadequate blinding, randomization, or incomplete control of confounding variables. In addition, a scarce amount of evidence was available to draw conclusions from regarding the efficacy of peer inclusion interventions on social functioning, which limited the translatability of the findings to practical settings. These methodological limitations were contributors to comparatively poorer Kmet ratings of the studies.

## 6. Conclusion

The limitations of pharmacological treatment make it necessary to investigate the use of psychosocial interventions such as peer inclusion interventions as an addition or alternative to medication. It is clear that interventions which include peer inclusion components may be an appropriate SST method for children with ADHD. However, RCTs and quasi-experimental studies of children with ADHD which meet all the criteria for peer mediation are needed. This meta-analysis found evidence of a substantial difference between peer inclusion interventions plus medication treatment versus medication alone. Peer inclusion interventions were significantly better at improving social competence and peer relations than no treatment or waitlist control groups, indicating that psychosocial interventions are valuable in treatment. There is a need for more studies to test the use of* peer-mediated* interventions; use typically developing peers; appropriately calculate sample size; and control for medication as a potential confound. In addition, the reporting on the specific characteristics and involvement of peers in the intervention, as demonstrated in the peer inclusion interventions for ASD research, will assist with clarity regarding methods, effectiveness, and outcomes. Furthermore, the current review systematically highlighted the necessity for more high quality studies to evaluate the use of peer inclusion interventions where the design allows for effect sizes to be calculated separately for peer, teacher, and parent components.

## Figures and Tables

**Table 1:** Full electronic search strategy.

	Database	Search terms	Limitations	Results
Subject headings	CINAHL	(MH “Attention Deficit Hyperactivity Disorder”) AND ((MH “Peer Group”) OR (MH “Peer Counseling”) OR (MH “Peer Review”) OR (MH “Friendship”) OR (MH “Psychotherapy, Group”) OR (MH “Group Processes”) OR (MH “Support Groups”) OR (MH “Role Playing”) OR (MH “Play Therapy”) OR (MH “Camps”) OR (MH “Social Skills Training”) OR (MH “Social Skills”) OR (MH “Communication Skills Training”) OR (MH “Students, High School”) OR (MH “Schools, Middle”) OR (MH “Schools, Special”) OR (MH “Schools, Secondary”) OR (MH “Schools, Elementary”) OR (MH “Social Behavior”))	Age: child, preschool: 2–5 years; child: 6–12 years; adolescent: 13–18 years	280
	Embase	(Attention deficit disorder/) AND (peer rejection/OR “peer review”/OR peer acceptance/or peer counseling/OR peer group/OR friendship/OR play/OR play therapy/OR group therapy/OR social adaptation/OR social behavior/OR social interaction/)	Age: preschool child <1 to 6 years>; school child <7 to 12 years>; adolescent <13 to 17 years> Language: English; Humans	1296
	Medline	(attention deficit disorder with hyperactivity/) AND (Friends/OR Psychotherapy, Group/OR play therapy/OR social behavior/)	Age: preschool child (2 to 5 years); child (6 to 12 years); adolescent (13 to 18 years) Language: English	505
	PsycINFO	(DE “Attention Deficit Disorder with Hyperactivity”) AND (DE (“peer counselling”) OR DE (“peer tutoring”) or DE (“peers”) OR DE (“peer evaluation”) OR DE (“friendship”) OR DE (“group intervention”) OR DE (“group participation”) OR DE (“group psychotherapy”) OR DE (“group dynamics”) OR DE (“group cohesion”) OR DE (“group participation”) OR DE (“childhood play behavior”) OR DE (“childhood play development”) OR DE (“play therapy”) OR DE (“therapeutic camps”) OR DE (“school based intervention”) OR DE (“psychosocial development”) OR DE (“psychosocial factors”) OR DE (“psychosocial readjustment”) OR DE (“psychosocial rehabilitation”) OR DE (“social skills”) OR DE (“social skills training”) OR DE (“social group work”) OR DE (“social groups”) OR DE (“social integration”) OR DE (“social interaction”) OR DE (“Social behaviour”) OR DE (“group counseling”))	Age: preschool age <age 2 to 5 yrs>; school age <age 6 to 12 yrs>; adolescence <age 13 to 17 yrs>	737

Free text words	CINAHL	(ADHD OR ADD OR “Attention deficit hyperactivity disorder”) AND (peer^*∗*^ OR friend^*∗*^ OR buddy OR buddies OR playmate^*∗*^ OR “group intervention^*∗*^” OR “group therap^*∗*^” OR “group role play^*∗*^” OR “play group^*∗*^” OR camp^*∗*^ OR stp OR “play-based” OR psychosocial OR school^*∗*^ OR “Social skills” OR “Social behavio^*∗*^”)	Published date: 20151101–20161131 Age: child, preschool: 2–5 years; child: 6–12 years; adolescent: 13–18 years	280
	Embase	*As per CINAHL Free Text*	Last year Age: preschool child <1 to 6 years>; school child <7 to 12 years>; adolescent <13 to 17 years> Field: Title and or Abstract	177
	Medline	*As per CINAHL Free Text*	Published date: 2016 - Current Age: preschool child (2 to 5 years); child (6 to 12 years); adolescent (13 to 18 years)	97
	PSYCINFO	*As per CINAHL Free Text*	Published date: 20151101–20161131 Age: preschool age <age 2 to 5 yrs>; school age <age 6 to 12 yrs>; adolescence <age 13 to 17 yrs> Field: Title and or Abstract	365

**Table 2:** Reasons for exclusion of papers published in peer reviewed journals.

Study	Reason for exclusion
Antshel [14]	No intervention
Arnett et al. [15]	No intervention
Erhardt and Hinshaw [16]	No intervention
Haas and Waschbusch [17]	No intervention
Landau and Moore [5]	No intervention
Mikami and Hinshaw [18]	No intervention
Mikami and Huang-Pollock [19]	No intervention

Antshel [14]	Not peer inclusion—description of intervention did not include peer inclusion component
Burrows [20]	Not peer inclusion—description of intervention did not include peer inclusion component
Charlebois [21]	Not peer inclusion—description of intervention did not include peer inclusion component
Evans and Schultz [22]	Not peer inclusion—description of intervention did not include peer inclusion component
Frame [23]	Not peer inclusion—description of intervention did not include peer inclusion component
Frame et al. [24]	Not peer inclusion—description of intervention did not include peer inclusion component
Gardner [25]	Not peer inclusion—description of intervention did not include peer inclusion component
Gerber et al. [26]	Not peer inclusion—description of intervention did not include peer inclusion component
Gerber-von Müller et al. [27]	Not peer inclusion—description of intervention did not include peer inclusion component
Langberg [28]	Not peer inclusion—description of intervention did not include peer inclusion component
Mikami et al. [29]	Not peer inclusion—description of intervention did not include peer inclusion component
Abdollahian et al. [30]	Not peer inclusion—study assessed ADHD symptoms with no social skills outcomes
Abikoff et al. [31]	Not peer inclusion—study assessed ADHD symptoms with no social skills outcomes
Antshel and Remer [32]	Not peer inclusion—study assessed ADHD symptoms with no social skills outcomes
Hariri and Faisal [33]	Not peer inclusion—study assessed ADHD symptoms with no social skills outcomes
Jans et al. [34]	Not peer inclusion—study assessed ADHD symptoms with no social skills outcomes
Jans et al. [35]	Not peer inclusion—study assessed ADHD symptoms with no social skills outcomes
Looyeh et al. [36]	Not peer inclusion—study assessed ADHD symptoms with no social skills outcomes
Tutty et al. [37]	Not peer inclusion—study assessed ADHD symptoms with no social skills outcomes

Burrows [20]	Peer inclusion—no social skills outcomes
DuPaul et al. [38]	Peer inclusion—no social skills outcomes
Gol and Jarus [39]	Peer inclusion—no social skills outcomes
Hechtman et al. [40]	Peer inclusion—no social skills outcomes
O'Connor et al. [41]	Peer inclusion—no social skills outcomes
Power et al. [42]	Peer inclusion—no social skills outcomes
Rickson and Watkins [43]	Peer inclusion—no social skills outcomes
Storebø et al. [44]	Peer inclusion—no social skills outcomes

Cantrill et al. [45]	No comparison group
Wilkes et al. [46]	No comparison group
Wilkes-Gillan et al. [47]	No comparison group
Wilkes-Gillan et al. [48]	No comparison group

Storebø et al. [49]	Protocol paper

Schmitman Gen Pothmann et al. [50]	Non-English
Tabaeian [51]	Non-English

*Note. *Table does not include seven excluded dissertations and one excluded conference abstract.

**Table 3:** Peer included studies for children with ADHD.

Design	Treatment condition	Participants N	Age Years (mean ± SD)	Inclusion criteria	Outcome measure^a^	Treatment outcome
Abikoff et al. [53] RCT: random assignment ADHD peer intervention	1 year weekly, 2nd year monthly (1) mph only (2) mph + MPT + SST (3) mph + ACT	mph: 34 (ADHD) mph + MPT: 34 (ADHD) mph + ACT: 35 (ADHD)	8.2 ± 0.8	ADHD diagnosis Positive response to mph	*Social skills outcomes*: SSRS: parent + child form *Others*: TOPS: teacher form Observations in gym	SSRS: significant improvement TOPS: significant improvement Significantly fewer positive and negative behaviours

Choi and Lee [54] RCT: random assignment ADHD peer intervention	Weekly EMT and SST treatment for 16 weeks (1) EMT group (2) SST group (3) Waitlist control	EMT: 25 SST: 25 Control: 24	EMT: 11.0 ± 0.9 SST: 11.1 ± 0.9 Control: 10.8 ± 0.8	ADHD diagnosis Total WISC-Revised Korean Version IQ > 90 Behaviour Problem Scale score in clinical range on CBCL	*Social skills outcomes*: Peer Relational Skills Scale *Others* Emotion Expression Scale for Children Child Depression Inventory State-Trait Anxiety Inventory for Children	No differences between SST and control groups. EMT group improved significantly more than control EMT group improved significantly more than SST and control groups SST group improved significantly more than control group, but no differences between EMT and control groups

Frankel et al. [55] QES: child and parent manually assigned Non-ADHD peer intervention	SST weekly for 12 weeks (1) Treatment (2) Waitlist control	Treatment 35: ADHD/14: no ADHD Waitlist control 12: ADHD/12 no ADHD	9.05 ± 3.06	Peer problems ADHD (using mph) ODD based on DSM-III-R	*Social skills outcomes*: SSRS: parent form; attention and self-control subscales *Others*: PEI: teacher form	SSRS: significantly greater improvement PEI: nonsignificant improvement on the withdrawal scale. Significant improvement on the aggression scale

Guli et al. [56] QES: children manually assigned ADHD and other diagnosis peer interventions	SST weekly for 12 weeks or twice weekly for 8 weeks (1) SCIP group (2) Clinical control	SCIP: 18 (5 ADHD/2 NLD/11 ASD) Control: 16 (3 ADHD/6 NLD/7 ASD)	10.97 ± 1.98	ADHD diagnosis Overall intelligence > 80 on WISC-III	*Social skills outcomes*: BASC: parent form; withdrawal and social skills subscales DANVA2 Observed social interaction SSRS: parent form *Others*: Parent and child interviews	BASC: no significant effects found DANVA2: no significant effects found Observations: medium effects for increases in positive interactions and decreases in solitary play for treatment group Baseline measure for presence of clinically significant social skills deficits 75% reported one or more specific positive changes

Haas et al. [57] QES: children manually assigned Non-ADHD peer intervention	Behavioural treatment for 8 weeks in the context of a STP (1) Treatment (2) Control	Treatment 54: ODD or CP/ADHD Control 16: no ODD or CP/ADHD	9.48 ± 1.58	ADHD diagnosis Nonmedicated	*Social skills outcomes*: SIRF: staff observations Peer sociometric interviews *Others*: Time-out measures	SIRF: significant improvement in social skills and problem solving Peer sociometrics: significant improvement in Likert and Dislike nominations Time-out: significant improvements

Hannesdottir et al. [58] RCT: random assignment ADHD peer intervention	Behavioural and SST treatment with working memory training (1) Treatment (2) Waitlist control (3) Parent training	Treatment: 16 Control: 14 Parent training: 11	9.2 ± 0.62	ADHD diagnosis	*Social skills outcomes*: SSRS: parent form *Others*: ADHD Rating Scale-IV ERC SDQ Icelandic WISC-IV	Significant group × time interactions favouring treatment group over waitlist control Significant group × time interactions favouring treatment group over waitlist control for inattention, but not hyperactivity/impulsivity No significant main effect of time No significant main effect of time Significant main effects on two subscales (Coding and Letter-Number Sequencing), but no significant group × time interactions

Hantson et al. [59] QES: child and parent manually assigned ADHD peer intervention	SST daily for 2 weeks in the context of an intensive therapeutic summer day camp (1) Treatment (2) Treatment as usual control	Treatment 33: ADHD Treatment as usual control 15: ADHD	8.6 ± 1.6	ADHD diagnosis IQ > 70 on WISC-III	*Social skills outcomes*: IPR: child form WFIRS-P: parent form *Others*: CGI-P: parent form	IPR: significant improvement WFIRS-P: Significant improvements except for WFIRS Risky Activities subscale CGI-P: significant improvement

Huang et al. [60] QES: child and parent manually assigned ADHD peer intervention	Weekly SST treatment for 8 weeks consisting of 80-minute sessions (1) SST group (2) No treatment control	SST: 45 Control: 52	SST: 8.2 ± 0.9 Control: 8.5 ± 0.9	ADHD diagnosis	*Social skills outcomes*: SSRS: child + teacher form *Others* SNAP: parent + teacher CBCL: child form	SSRS-C: significant improvement in Self-Control in favour of SST group; SSRS-T: significant improvement in active participation in favour of SST group SNAP-P: main effect of group on Oppositional subscale; SNAP –T: Main effect of group on Active Participation subscale CBCL-C: main effect of group on Anxious/Depressed subscale

Jensen et al. [61] RCT: random assignment 3-year follow-up ADHD peer intervention	Treatment over 14 months (1) Medication only (2) Behavioural treatment incl. parent training + STP + school-based treatment (3) Combined incl. medication + behavioural treatment (4) Community care control	Medication management 115: (ADHD) Behavioural treatment 127: (ADHD) Combined treatment 127: (ADHD) Community care control 116: (ADHD)	11.8 ± 0.95	Children who participated in the 1999 MTA study	*Social skills outcomes*: SSRS: parent + teacher forms *Others*: SNAP: parent + teacher forms WIAT: reading score CIS	SSRS: effect size for improvement from baseline to 36 months across all treatment groups was 0.8–0.9 SNAP: effect size for improvement from baseline to 36 months across all treatment groups was 1.6–1.7 for ADHD and 0.7 for ODD WIAT: effect size for improvement from baseline to 36 months across all treatment groups was 0.1–0.2 CIS: effect size for improvement from baseline to 36 months across all treatment groups was 0.9–1.0

Kolko et al. [62] QES: children manually assigned ADHD and other diagnosis peer interventions	3 weekly sessions for 5 weeks (1) SCST group (2) SA group	SCST: 36 (10 ADHD/11 CD/15 OD) SA: 20 (4 ADHD/12 CD/4 OD)	10.4 ± 2.1	Score of at least 7 on a four-item social problems screen, with at least one maximum rating	*Social skills outcomes*: CAI-M: Self-report LNS-M: Self-report SPS: Staff report Sociometric Ratings: Staff Peer Nomination Behavioural Role-Play In vivo behavioural observations	CAI-M: significant improvement in post-training scores LNS-M: SCST group showed significant reduction in post-training scores SPS: SCST group showed significant reduction in posttraining scores Both groups improved significant SCST group showed greater pre-post reduction nominations SCST group showed significant improvement SCST group exhibited significant improvement

Mikami et al. [63] RCT: random assignment Typically developing peer intervention	Weekdays for four weeks total Weekdays for 2 weeks per treatment allocation (1) MOSAIC then COMET (2) COMET then MOSAIC	MOSAIC: 12 ADHD/58 TD COMET: 12 ADHD/55 TD	8.15 ± 0.79	ADHD diagnosis after screening IQ >80 on WASIl	*Social skills outcomes*: Peer sociometric nominations Peer interaction observations Messages from peers *Others*: Teacher-Child Rating Scale subscales of problem behaviours	Main effect on positive nominations for treatment was not significant. Received fewer negative nominations and more reciprocated friendship nominations when in MOSAIC relative to COMET group No main effect for treatment condition Received a significantly greater proportion of positive messages when in MOSAIC relative to COMET group No main effects for treatment condition

MTA Cooperative Group [64] RCT: random assignment ADHD peer intervention	Treatment over 14 months (1) Medication only (2) Behavioural treatment incl. parent training + STP + school-based treatment (3) Combined incl. medication + behavioural treatment (4) Community care control	Medication management 144: (ADHD) Behavioural treatment 144: (ADHD) Combined treatment 145: (ADHD) Community care control 146: (ADHD)	8.5 ± 0.8	ADHD combined type diagnosis In residence with the same primary caretaker(s) for last 6 months or longer	*Social skills outcomes*: SSRS: parent + teacher *Others*: SNAP: parent + teacher MASC: child form Parent-child relationship questionnaire WIAT: (reading, math + spelling)	SSRS: significant improvement for parent-reported internalizing problems for combined treatment over behavioural treatment SNAP: combined + medication management were clinically and statistically superior to behavioural treatment + community care MASC: improvements of small magnitude Improvements of small magnitude Significant improvement for reading achievement score for combined treatment over behavioural treatment

MTA Cooperative Group [65] RCT: random assignment 24-month follow-up ADHD peer intervention	Treatment over 14 months (1) Medication only (2) Behavioural treatment incl. parent training + STP + school-based treatment (3) Combined incl. medication + behavioural treatment (4) Community care control	Medication management 128: (ADHD) Behavioural treatment 139: (ADHD) Combined treatment 138: (ADHD) Community care control 135: (ADHD)	8.4 ± 0.8	Children who participated in the 1999 MTA study	*Social skills outcomes*: SSRS: parent + teacher *Others*: SNAP: parent + teacher Negative/ineffective discipline factor WIAT: (reading, math + spelling)	SSRS: nonsignificant overall treatment effect SNAP: significant overall treatment effect Nonsignificant overall treatment effect WIAT: nonsignificant overall treatment effect

Pfiffner et al. [66] RCT: random assignment ADHD and other diagnosis peer interventions	Treatment over 12 weeks First cohort: (1) CLAS program (2) Waitlist control Second-Fifth cohort: (1) CLAS program (2) Treatment as usual control	Five cohorts of children randomized to either CLAS program or control 25: ADHD, 2: Undifferentiated Attention Deficit Disorder; 19: Oppositional Defiant Disorder, 3: Conduct Disorder, 4: Separation Anxiety Disorder, 5: Overanxious Disorder, 2: Dysthymic Disorder.	8.7 ± 1.2	ADHD diagnosis IQ > 80 on the WASI Attending school full time with school consenting to participate in school-based treatment	*Social skills outcomes*: SSRS: parent + teacher *Others*: Child Symptom Inventory SCT Scale COSS: parent + teacher Test of Life Skill Knowledge Clinical Global Impressions: parent + teacher	SSRS: significant improvements Significant reductions in number of DSM-IV inattention symptoms SCT: significant treatment effects COSS: significant improvements of organisational skills Significantly improvements Significantly greater improvement

Shechtman and Katz [67] RCT: random assignment ADHD and other diagnosis peer interventions	Weekly for 15 weeks (1) Group therapy (2) Waitlist control	Group Therapy: 42 (20: ADD or ADHD/22 LD) Waitlist control: 45 (14 ADD or ADHD/31 LD)	13.26 ± 0.77	ADD/ADHD or LD diagnosis	*Social skills outcomes*: Adolescent Interpersonal Competence Questionnaire Intimate Friendship Scale Working Alliance Inventory	Significant treatment condition by time effect Nonsignificant treatment condition by time effect High association between bonding with group members and gains on social competence

Storebø et al. [68] RCT: random assignment ADHD peer intervention	Weekly for 8 weeks (1) Experimental treatment incl. SST + parent training + standard treatment (2) Standard treatment alone	Experimental treatment 28: ADHD Standard treatment alone 27: ADHD	10.4 ± 1.31	ADHD diagnosis IQ > 80 Not previously medicated	Indexes from Conners 3 and Connors CBRS: *Social skills outcomes*: Social Problems score Peer Relations score *Others*: Executive Functions score Academic score Aggressiveness score Emotional score Hyperactivity score	No statistically significant difference when comparing groups Neutral result between groups Neutral result between groups Neutral result between groups Highly significant changes towards fewer symptoms No statistically significant difference when comparing groups

Waxmonsky et al. [69] RCT: random assignment ADHD peer intervention	Weekly for 8 weeks (1) Atomoxetine + BT (2) Atomoxetine alone	Atomoxetine + BT 29: ADHD Atomoxetine alone 27: ADHD	8.59 ± 1.58	ADHD diagnosis IQ > 75 Positive response to atomoxetine	*Social skills outcomes*: SSRS: parent + teacher *Others*: Student Behaviour Teacher Response Observation Code DBD: parent + teacher APRS: teacher PSERS: parent + teacher CDRS-R: child + parent interview DRC/ITBE Clinical Global Impressions Scale: clinician	SSRS: significantly lower parent-rated problem behaviours No difference between groups post-treatment DBD: marginally significant improvement of ADHD and ODD symptoms APRS: significantly higher teacher-rated impulse control PSERS: mean scores well within the mild range. Marginally lower depression scores post-treatment. Suicidal thoughts decreased significantly over time but with no difference between groups DRC/ITBE: Significant main effect of medication/time 51.9% of subjects in Atomoxetine-only and 55.2% of Atomoxetine + BT subjects were rated as much or very much improved

*Notes. *RCT = randomized controlled trial, QES = quasi-experimental study, mph = methylphenidate, MPT = Multipsychosocial Treatment, SST = social skills training, ACT = attention control treatment, ADHD = Attention-Deficit Hyperactivity Disorder, SSRS =Social Skills Rating Scale [70], TOPS =Taxonomy of Problem Situations [71], EMT = emotional management training, WISC-Revised Korean Version = Wechsler Intelligence Scale for Children-Revised Korean Version [72], CBCL = Child Behaviour Checklist [73, 74], ODD = oppositional defiant disorder, DSM-III-R = Diagnostic and Statistical Manual of Mental Disorders 3rd Edition, PEI = Pupil Evaluation Inventory [75], SCIP = Social Competence Intervention Program, NLD = nonverbal learning disorder, ASD = autism spectrum disorder, WISC-III (Weschler Intelligence Scale for Children-III [76]), BASC = Behaviour Assessment System for Children [77], DANVA2 = Diagnostic Analysis of Nonverbal Accuracy 2 Nowicki, 2004, CP = conduct problems, SIRF = Staff Improvement Rating Form [78], ERC = Emotion Regulation Checklist [79]; SDQ = Strengths and Difficulties Questionnaire [80], Icelandic WISC-IV = Wechsler Intelligence Scale for Children-Icelandic version [81], IPR = Index of Peer Relations [82], WFIRS-P = Weiss Functional Impairment Rating Scale-Parent Version [83], CGI-P = Conners' Global Index-Parent Version [84], SNAP = (Swanson, Nolan, and Pelham Rating Scale [85], STP = summer treatment program, MTA = Multimodal Treatment Study of Children with ADHD, WIAT = Wechsler Individual Achievement Test [86], CIS = Columbia Impairment Scale [87], SCST = social-cognitive skills training, SA = social activity group, CD = conduct disorder, OD = other disorders, CAI-M = Children's Assertiveness Inventory-Modified [88], LNS-M = Loneliness Scale for Children-Modified [89], SPS = Social Problems Screen, MOSAIC = Making Socially Accepting Inclusive Classrooms, COMET = contingency management training, TD = typically developing, WASI = Wechsler Abbreviated Scale of Intelligence [90], MASC = Multidimensional Anxiety Scale for Children [91], CLAS = Child Life and Attention Skills program, SCT Scale = Sluggish Cognitive Tempo Scale [92], COSS = Children's Organizational Scale [93], DSM-IV = Diagnostic and Statistical Manual of Mental Disorders 4th Edition, LD = learning disabilities, Conners 3 [94], Conners CBRS = Conners Behaviour Rating Scales [94], BT = behaviour therapy, DBD = Disruptive Behaviour Disorders Rating Scale [95], APRS = Academic Performance Rating Scale [96], PSERS = Pittsburgh Side Effects Rating Scale [97], CDRS-R = Children's Depression Rating Scale-Revised [98], and DRC/ITBE = Daily Report Card/Individual Target Behaviour Evaluation. ^a^When handling studies with multiple social skills outcome measures, one singular outcome measure was chosen that most comprehensively reflected the construct social skills. This singular outcome measure was then used for the calculation of an effect size. No measures were aggregated within the study to obtain one effect size.

**Table 4:** Intervention components of included studies.

Study	Peer component	Parent component	Teacher component	Skills	Medication
Frankel et al. [55] QES	Children were didactically presented social skills and required to rehearse behaviours between each other. Participants were also taught conversational techniques and rehearsed them in the context of introductions to other class members	(i) Parent sessions (ii) Parent ratings of social skills (iii) Child socialisation homework	(i) Teacher ratings of antisocial, prosocial, and aggressive behaviour	(i) Conversation (ii) Techniques (iii) Playing together/getting along (iv) Giving compliments & criticism	All ADHD participants were required to take medication (incl. methylphenidate, dextroamphetamine, pemoline, other psychotropic medication)

Guli et al. [56] QES	The sessions included activities that focus on establishing social skills through several improvisations or process dramas, through which they practice perspective taking and cognitive flexibility with their peers.	(i) Parent ratings of social skills (ii) Parents encouraged home challenges	(i) None	(i) Group cohesion (ii) Emotional knowledge (iii) Focusing attention (iv) Facial expression (v) Body language (vi) Vocal cues (vii) Nonverbal cues	51.3% of participants were reported to take prescription medication. Treatment: 12 medication & 6 no medication; Control: 4 medication & 12 no medication

Haas et al. [57]	Counsellor-led questions prompted a discussion of the social skills by encouraging children to provide a description of the social skills (e.g., definition, examples) and to model and role-play good and bad examples of how to use the social skill.	(i) Parent ratings of ADHD, ODD, and CD symptoms (ii) Parent ratings of callous/unemotional traits	(i) None	Social skills: (i) Validation (ii) Cooperation (iii) Communication (iv) Participation	All ADHD participants were either not taking medication or prescribed a placebo assignment. However, some children were on medication for some (but not all) days during the summer treatment program

Hantson et al. [59] QES	The therapist first described how to perform the skills in an appropriate manner. The children were then paired and asked to role-play the new skill in front of the group. Following this, children were asked to role-play the skills from the other's perspective in an effort to understand situations from other person's point of view.	(i) Parent psychoeducation and training (ii) Parent ratings of function, behaviour, and ADHD symptoms	(i) None	Social skills: (i) Introducing self (ii) Joining in (iii) Knowing your feelings (iv) Dealing with anger (v) Self-control (vi) Responding to teasing (vii) Staying out of fights	Participants who were on medication stayed on medication; those who were not on medication remained so. Treatment: 20 medication & 13 no medication; control: 9 medication & 6 no medication

Huang et al. [60] QES	Children were taught various social skill modules via didactic instructions, modelling, role-play activities and behavioural rehearsals Positive social behaviour was reinforced via a token system.	(i) Weekly parent sessions to educate on ADHD (ii) Parent ratings of social skills	Teacher ratings of attention, hyperactivity, impulsivity, oppositional, cooperative behaviour, self-assertion, self-control and conflict coping	(i) Conversation (ii) Playing together/getting along (iii) Giving compliments & criticism	All ADHD participants received methylphenidate with drug compliance controlled

Kolko et al. [62] QES	Cotherapists and children engaged in several role-plays. The group discussed each role-play and provided constructive performance feedback. Inadequate role-plays were rehearsed a second time to promote mastery.	(i) None	(i) One-year follow-up teacher ratings of social skills and outcome measures	(i) Social involvement (ii) Gaze (iii) Physical space (iv) Voice volume/inflection Openers/compliments (v) Positive assertion (vi) Negative assertion (vii) Appropriate nonaggressive play or sharing	Comparable percentages of children in SCST and SA groups received methylphenidate (22% versus 20%), imipramine (11% versus 10%), lithium (8% versus 5%), or other medications (7% versus 5%)

Abikoff et al. [53] RCT	Peers role-played and modelled appropriate and inappropriate social behaviours in groups of four.	(i) Parent training (ii) Parent ratings of social skills	(i) Teacher ratings of socially rejected and accepted children (ii) Reinforcement strategies, daily school report card	(i) Basic interaction skills (ii) Getting along with others (iii) Contacts with adults at home and school (iv) Conversation skills (v) Problem situations	All participants were prescribed methylphenidate after a 5-week clinical methylphenidate trial and placebo substitution to determine positive response to medication prior to treatment

Choi and Lee [54] RCT	EMT: children undertook activities that covered four major behavioural characteristics: (1) identification and labelling of emotional words; (2) emotional recognition and expression; (3) emotional understanding; and (4) emotional regulation in social situations SST: children were taught various social skills to improve their interactions with peers and teachers by using prompts, role-play and reinforcement	(i) Parent ratings on emotional and behavioural problems in children	Interacted with children as part of the SST and EMT programs	(i) Basic interaction skills (ii) Regulating emotions within a group (iii) Problem-solving skills (iv) Conversation skills (v) Listening skills (vi) Reaction to rejection, negotiation, being teased and criticised	All participants were prescribed with medication during the course of the study although not controlled

Hannesdottir et al. [58] RCT	Therapists lead discussions amongst groups of three children to aid solving problems presented at a number of “stations.” Stations included the Emotion Station, Friendship Station, Stopping Station, and Problem-Solving Station. In addition, there was a Brain Training Station, at which children practiced computer-based executive function tasks.	(i) Parent ratings of social skills (ii) Parent training (one meeting)	None	(i) Identifying facial expressions (ii) Hiding feelings (iii) Relaxation and anger management techniques (iv) Interpreting ambiguous situations (v) Meeting new peers (vi) Reading nonverbal messages (vii) Compromising (viii) Working memory (ix) Thinking before acting/speaking (x) Problem solving everyday problems	100% of participants in treatment group were on medication for the duration of the study. There were 12 participants on medication in the control group (85.7%) at study commencement, dropping to 11 participants at the end of the study (78.6%)

Jensen et al. [61] RCT	Sessions include instruction, modelling, role-playing and practice in key social concepts such as communication, as well as more specific skills. In addition to these sessions, the children engaged in a daily cooperative group task that is designed to promote cooperation and contribute to cohesive peer relationships. A buddy system was employed to help children develop individual friendships that may “buffer” them from the possible negative effects of being unpopular. This was accomplished by assigning each child a buddy with whom their goal is to form a close friendship. The children engage in a variety of activities with their buddies and meet regularly with adult “buddy coaches” who assist them in working out relationship problems.	(i) Parent training (ii) Parent ratings of ADHD, internalizing, oppositional, and aggressive symptoms, and social skills	School-based treatment: (i) Teacher consultation focused on behaviour management strategies (ii) Paraprofessional aid (iii) Teacher ratings of ADHD, internalizing, oppositional, and aggressive symptoms and social skills	(i) Social skills effective for peer group functioning	71% of combined treatment and medication management participants were using medication at high levels compared to 62% and 45% of community care and behavioural treatment participants, respectively. Average medication doses differed across all groups

Mikami, Griggs [63] RCT	Peers were trained to be more socially inclusive in the MOSAIC treatment condition. Teachers assigned children to work in teams for collaborative activities where children had to work together in order to succeed.	(i) None	(i) Summer program teacher ratings of problem behaviours	(i) Social skills (ii) Social inclusion (iii) Peer group functioning	10 out of 24 children with ADHD were medicated with psychotropic medication, and some were taking additional medications for comorbid conditions. All medicated children stayed on a consistent regimen during the summer program

The MTA Cooperative Group [64] RCT	Sessions included instruction, modelling, role-playing, and practice in key social concepts such as communication, as well as more specific skills. In addition to these sessions, the children engaged in a daily cooperative group task that was designed to promote cooperation and contribute to cohesive peer relationships. A Buddy System was employed to help children develop individual friendships that may “buffer” them from the possible negative effects of being unpopular. This was accomplished by assigning each child a buddy with whom their goal is to form a close friendship. The children engaged in a variety of activities with their buddies and met regularly with adult “buddy coaches” who assisted them in working out relationship problems.	(i) Parent training (ii) Parent ratings of ADHD, internalizing, oppositional, and aggressive symptoms, and social skills	School-based treatment: (i) Teacher consultation focused on behaviour management strategies (ii) Paraprofessional aid (iii) Teacher ratings of ADHD, internalizing, oppositional, and aggressive symptoms, and social skills	(i) Social skills effective for peer group functioning	All participants in the treatment groups were prescribed medication, however 3.1% of the combined treatment and medication management subjects were on no medication

MTA Cooperative Group [65] RCT	Sessions included instruction, modelling, role-playing, and practice in key social concepts such as communication, as well as more specific skills. In addition to these sessions, the children engaged in a daily cooperative group task that was designed to promote cooperation and contribute to cohesive peer relationships. A Buddy System was employed to help children develop individual friendships that may “buffer” them from the possible negative effects of being unpopular. This was accomplished by assigning each child a buddy with whom their goal is to form a close friendship. The children engaged in a variety of activities with their buddies and met regularly with adult “buddy coaches” who assisted them in working out relationship problems.	(i) Parent training (ii) Parent ratings of ADHD, internalizing, oppositional, and aggressive symptoms, and social skills	School-based treatment: (i) Teacher consultation focused on behaviour management strategies (ii) Paraprofessional aid (iii) Teacher ratings of ADHD, internalizing, oppositional, and aggressive symptoms, and social skills	(i) Social skills effective for peer group functioning	70% of combined treatment and 72% of medication management participants were using medication at high levels compared to 62% and 38% of community care and behavioural treatment, respectively

Pfiffner et al. [66] RCT	Children role-played the positive use of a skill, using brief scripts of common problem situations with peers or siblings (e.g., entering a game, getting out during a game, and being teased). Children evaluated each other's performance of the social skills immediately after each role-play and were called on to give specific reasons for their ratings.	(i) Parent training (ii) Parent ratings of inattention and sluggish cognitive tempo symptoms, social skills, organizational skills, and overall improvement	(i) Teacher consultation (ii) School-home daily report card (iii) Teacher ratings of inattention and sluggish cognitive tempo symptoms, social skills, organizational skills, and overall improvement	(i) Social competence (ii) Academic (iii) Study (iv) Organization (v) Self-care (vi) Daily living skills	Children were excluded if they changed medication status during the course of the study. Only two subjects (both in CLAS program group) began the study taking medication (atomoxetine); they continued medication at posttreatment and follow-up. Two children in the control group began medication at posttreatment, and one did so at follow-up

Shechtman and Katz [67] RCT	The expressive-supportive modality uses an integrative theoretical approach in therapy, with a strong emphasis on self-expressiveness and group support. Activities and therapeutic games are consistently used to help participants function in the group process.	(i) None	(i) None	(i) Initiation (ii) Emotional support (iii) Negative assertion (iv) Disclosure (v) Coping with conflicts (vi) Intimacy in friendship	No mention of whether participants were medicated or nonmedicated

Storebø et al. [68] RCT	Different methods of teaching the children were used. These include didactic instructions, work with symbols (e.g., dolls), role-play, creative techniques, physical exercises, music, story reading, games, and movies. Each session had a theme of a particular aspect of social skills training.	(i) Parent training (ii) Parent educational group (iii) Parental screen for adult ADHD symptoms	(i) Teacher ratings of academic and behavioural performance, social problems, peer relations and emotional regulation	(i) Self-worth (ii) Nonverbal communication (iii) Feelings (iv) Impulse control (v) Aggression management (vi) Conflict resolution (vii) Problem solving (viii) Social cues	All participants were prescribed medication. Treatment started with the first choice: methylphenidate; the second choice: dexamphetamine; and atomoxetine if significant anxiety component change or suspicion of dexamphetamine abuse

Waxmonsky et al. [69] RCT	Each session began with a brief description of the social skills of the day, which was presented to the child didactically and through modelling and role-playing.	(i) Parent training (ii) Parent ratings of ADHD, ODD, CD, and depression symptoms, social skills, problem levels, adverse emotional events	(i) Teacher implemented daily report card (ii) Teacher ratings of academic and behavioural performance, and adverse emotional events	(i) Cooperation (ii) Participation (iii) Validation (iv) Communication (v) Following rules (vi) Completing assignments (vii) Complying with adults (viii) Teasing	All participants were prescribed atomoxetine If a subject was already taking ADHD medication other than atomoxetine, the other medication was stopped for at least 48 hours prior to screening

*Notes*. QES = quasi-experimental study; RCT = randomized controlled trial; ADHD = attention-deficit/hyperactivity disorder; ODD = oppositional defiant disorder; CD = conduct disorder; SCST = social-cognitive skills training; SA = social activity; EMT = emotional management training; SST = social skills training; MOSAIC = Making Socially Accepting Inclusive Classrooms; and CLAS = Child Life and Attention Skills program.

**Table 5:** Methodological quality of included studies.

Study	Treatment	Control	NHMRC evidence level	Randomization	Blinding	Methodological quality
Abikoff et al. [53]	Child and parent training with medication	Attention control with medication	II	Block randomization scheme with blocks of 4 children. The groups were balanced for age, sex, oppositional defiant disorder, and ethnicity	Trained observers, blind to treatment and diagnosis, observed the study children and classmates as a primary outcome	Strong quality (score 23/28). Reliable use of peers. Sampling strategy appropriate with subject characteristics sufficiently described in companion methodological article presented by Klein et al. (2004). Insufficient data to assess sample size. Blinding for one of the primary outcomes was reported. Randomization not reported in detail. No estimates of variance reported.

Choi and Lee [54]	Child training	Waitlist control	II	Block randomization scheme with blocks of 5 children. No evidence of stratification	No blinding of participants or personnel reported. All instruments were either parent- or child-report measures	Good quality score (18/28). Reliable use of peers. Sampling strategy appropriate with but only subject age, gender and school grade described. Blinding not reported for outcome measurement. Insufficient data to assess sample size. Estimates of variance provided for time points of interest, but not around the difference.

Frankel et al. [55]	Child and parent training with medication	Waitlist control with medication	III	No randomization evident	No blinding of participants or personnel reported	Good quality (score 18/28). Reliable use of peers. Sampling strategy appropriate with subject characteristics sufficiently described. Insufficient data to assess sample size. Blinding and randomization not reported. Authors reported the treatment and waitlist groups differed significantly in mean socioeconomic status however did not correlate with any outcome variable. Authors reported ADHD children were prescribed medication by their own private physicians and dosage was not verified by the present authors thus possibly affecting the results.

Guli et al. [56]	Child training	Clinical control	III	No randomization evident	No blinding of participants or personnel reported	Good quality (score 20/28). Reliable use of peers. Sampling strategy appropriate with subject characteristics sufficiently described. Insufficient data to assess sample size. Blinding and randomization not reported. Estimates of variance provided. Results reported in sufficient detail with supporting conclusions. Authors reported some children in the treatment and control group were taking prescription medication thus possibly affecting the results.

Haas et al. [57]	Child training	Child training for no diagnosis control	III	No randomization evident	Counsellors who rated children's behaviour were naïve to the conduct problems and callous/unemotional traits status of each child	Good quality (score 20/28). Reliable use of peers. Sampling strategy not described. Subject characteristics sufficiently described. Blinding reported for one of the primary outcomes. Randomization not reported. Authors reported a relatively small sample with limited power to detect trends. As the sample only included children with high levels of CP and ADHD the data can only be safely generalised to children with high levels of CP and ADHD. Treatment outcomes may have been affected by medicated and unmedicated behaviour as some children were on medication for some (but not all) days therefore this may have affected results.

Hannesdottir et al. [58]	Child training	Waitlist control	III	Randomization procedure not described	No blinding of participants or personnel reported	Good quality score (18/28). Reliable use of peers. Sampling strategy appropriate with subject characteristics sufficiently described. Randomization was reported, but procedure not described. Blinding not reported. Insufficient information to calculate sample size. No estimates of variance around differences reported.

Hantson et al. [59]	Child and parent training	Treatment as usual control	III	No randomization evident	No blinding of participants or personnel reported	Good quality (score 19/28). Reliable use of peers. Sampling strategy appropriate with subject characteristics sufficiently described, however comorbidities were not reported. Blinding and randomization not reported. Relatively small sample size. No estimates of variance reported.

Huang et al. [60]	Child and parent training	Community care control	II	No randomization evident	No blinding of participants or personnel reported	Good quality score (17/28). Reliable use of peers. Sampling strategy appropriate with subject characteristics sufficiently described. Control group consisted of families interested in the program but unable to attend at specific appointment times, thus not truly randomized. No blinding reported. Insufficient information to calculate sample size. No estimates of variance around differences reported. Authors recognise that participants with good medication compliance had better outcomes on some measures, but did not control for medication compliance in all analyses.

Jensen et al. [61]	Child, parent, and school-based training with medication	Community care control	II	Randomization was done centrally and stratified by site in blocks of 16 (4 to each group). Sealed, ordered envelopes were sent to sites for successive entries. Treatment assignment was concealed until the family confirmed agreement to accept randomization	No blinding of personnel reported	Good quality (score 21/28). Reliable use of peers. Sampling strategy appropriate with subject characteristics sufficiently described. Blinding was reported however it is not clear who was blinded. Randomization not described in detail. Appropriate samp**l**e size. Variance estimates reported inappropriately as study provided variance around the parameters of interest however not around the difference.

Kolko et al. [62]	Child training	Child socialisation group	III	No randomization evident	Second trained research assistant unaware of child's group assignment recorded in vivo behavioural observations. Interrater agreement of behavioural role-play test were assessed by comparing ratings assigned by a trained research assistant unaware of group assignment	Good quality (score 18/28). Reliable use of peers. Sampling strategy may have introduced bias. Subject characteristics sufficiently described. Blinding was adequately reported for some of the raters however not all. Randomization was not reported. No estimates of variance reported. Appropriate sample size.

Mikami et al. [63]	Child training for ADHD children	Child training for TD children	II	Randomly assigned via a computer-generated sequence either to a classroom in the MOSAIC treatment condition in Session 1 and a different classroom in the COMET treatment condition in Session 2 or vice versa. Assignment was stratified by child age and sex	Trained research assistants unaware of treatment group administered all primary outcome measures	Strong quality (score 25/28). Reliable use of peers. Sampling strategy appropriate with subject characteristics sufficiently described. Blinding was adequately reported for all outcome measures. Randomization was adequately reported. Estimates of variance provided. Authors reported a small sample size which limits the confidence for moderation results.

The MTA Cooperative Group [64]	Child, parent, and school-based training with medication	Community care control	II	Randomization was done centrally and stratified by site in blocks of 16 (4 to each group). Sealed, ordered envelopes were sent to sites for successive entries. Treatment assignment was concealed until the family confirmed agreement to accept randomization	The open parent, teacher, and child ratings for 5 out of 6 outcomes were augmented by blinded ratings of school-based ADHD and oppositional/aggressive symptoms. Raters blind to treatment condition performed standardized laboratory tasks to assess parent-child interactions	Strong quality (score 24/28). Reliable use of peers. Sampling strategy appropriate with subject characteristics sufficiently described. Blinding was adequately reported. Randomization not reported in detail. Appropriate sample size based on power analyses. Explicit use of manualised, evidence-based treatments and comprehensive range of outcome assessments. Variance estimates reported inappropriately as study provided variance around the parameters of interest however not around the difference.

MTA Cooperative Group [65]	Child, parent, and school-based training with medication	Community care control	II	Randomization was done centrally and stratified by site in blocks of 16 (4 to each group). Sealed, ordered envelopes were sent to sites for successive entries. Treatment assignment was concealed until the family confirmed agreement to accept randomization	No blinding of personnel reported	Good quality (score 21/28). Reliable use of peers. Sampling strategy appropriate with subject characteristics sufficiently described. Blinding not reported. Randomization not described in detail. Appropriate samp**l**e size. Variance estimates reported inappropriately as study provided variance around the parameters of interest however not around the difference.

Pfiffner et al. [66]	Child and parent training with teacher consultation	Waitlist control or treatment as usual	II	Randomization was stratified by sex (when two children of the same sex were identified, one was randomly assigned to CLAS program and one to the control group). Investigators required at least two participants of the same sex in the treatment group	Interviewers and raters who were blind to child's group assignment administered the Test of Life Skill Knowledge outcome measure	Good quality (score 22/28). Reliable use of peers. Sampling strategy appropriate with subject characteristics sufficiently described. Blinding was adequately reported. Randomization was not reported in detail. No estimates of variance reported. Appropriate sample size. Participant use of medication was a confounding variable however unlikely to have seriously distorted results.

Shechtman and Katz [67]	Child training for ADHD children	Waitlist control	II	The group was randomly divided (by alphabetical order) into an experimental and a waitlist control group (to be treated the next year)	No blinding of participants or personnel reported	Strong quality (score 24/28). Reliable use of peers. Sampling strategy appropriate with subject characteristics sufficiently described. Blinding not reported. Randomization was adequately reported. Estimates of variance provided. Appropriate sample size. Participant use of medication was not reported therefore may be confounding.

Storebø et al. [68]	Child and parent training with standard treatment	Standard treatment alone	II	The Copenhagen Trial Unit conducted central randomization with computer-generated, permuted randomization sequences in blocks of four with an allocation ratio of 1 : 1 stratified for sex and comorbidity	The interventions given were not “blind” to participants, parents, treating physicians, or personnel. However, the outcome assessors (teachers) were kept blinded of the allocated intervention. Blinded data were then handed over for data entry and statistical analyses	Strong quality (score 26/28). Reliable use of peers. Sampling strategy appropriate with subject characteristics sufficiently described. Blinding was adequately reported for all outcome measures. Blinding was not apparent for participants. Randomization was reported in detail. Estimates of variance provided. Sample size calculation completed with an appropriate sample size collected.

Waxmonsky et al. [69]	Child and parent training with school-based daily report card and medication	Medication alone	II	One-half of the subjects were randomly assigned to receive atomoxetine + BT and the remaining subjects randomly assigned to receive atomoxetine alone	The second (reliability) observer for the behaviour therapy arm was blinded to group assignment	Good quality (score 22/28). Reliable use of peers. Sampling strategy appropriate with subject characteristics sufficiently described. Primary investigators were not blinded. Randomization was not reported in detail. Variance estimates reported inappropriately as study provided variance around the parameters of interest however not around the difference. Sample size was calculated for the primary outcome measure however not the secondary measures therefore sample may not have had sufficient subjects to detect group differences for secondary measures.

*Notes*. NHMRC level II = RCTs; level III = quasi-experimental designs without random allocation; ADHD = Attention-Deficit Hyperactivity Disorder; CP = conduct problems; MOSAIC = Making Socially Accepting Inclusive Classrooms; COMET = contingency management training; and CLAS = Child Life and Attention Skills program.

**Table 6:** Subgroup analysis comparing intervention groups of included studies.

Subgroups	Hedges' *g*	*Z*-value	*p *value
*Parent component*	0.595	9.085	<0.001^*∗*^
No parent involvement (*N* = 4)	0.606	4.606	<0.001^*∗*^
Parent involvement (*N* = 18)	0.577	7.833	<0.001^*∗*^
*Study design*	0.629	11.344	<0.001^*∗*^
RCT (*N* = 16)	0.643	11.025	<0.001^*∗*^
Quasi-experimental (*N* = 6)	0.496	2.784	0.001^*∗*^
*Methodological quality*	0.609	5.696	<0.001^*∗*^
Good (*N* = 17)	0.576	7.206	<0.001^*∗*^
Strong (*N* = 5)	0.588	3.712	<0.001^*∗*^
*Blinding of outcome measures*	0.608	11.176	<0.001^*∗*^
Blinded (*N* = 12)	0.622	10.187	<0.001^*∗*^
No blinding (*N* = 10)	0.556	4.623	<0.001^*∗*^
*Outcome measure respondent type*	0.594	9.999	<0.001^*∗*^
Parent + teacher (*N* = 3)	0.832	6.786	<0.001^*∗*^
Parent (*N* = 6)	0.496	4.689	<0.001^*∗*^
Self (*N* = 6)	0.517	5.023	<0.001^*∗*^
Teacher (*N* = 7)	0.547	5.023	<0.001^*∗*^

*Notes.* ^*∗*^Significant.

**Table 7:** Metaregression results.

Set	Covariate	95% lower	95% upper	*Z*-value	2-sided *p* value
	Intercept	−0.0527	2.0452	1.86	0.0627
	Blinding: no blinding	−0.5548	0.158	−1.09	0.2752
	Parent Component: parent involvement	−0.5257	0.4463	−0.16	0.8729
	Methodological quality: strong quality	−0.5003	0.5989	0.18	0.8603
	Study design: RCT	−0.5616	0.6237	0.10	0.9182
*Rater*	Rater: parent-rated	−0.9472	−0.0091	−2.00	0.0457^*∗*^
*Rater*	Rater: self-rated	−0.959	0.4601	−0.69	0.4908
*Rater*	Rater: teacher- rated	−1.0655	0.1701	−1.42	0.1555

*Notes.* ^*∗*^Significant.

## Additional Points

References [53–69] are the studies included in the review while all the other references are additional references.

## Supplementary Materials

Supplementary Materials Supplementary Table  1: the methodology and reporting of this systematic review and meta-analysis in accordance with The PRISMA 2009 Checklist.
